# Delivering a “Dose of Hope”: A Faith-Based Program to Increase Older African Americans’ Participation in Clinical Trials

**DOI:** 10.2196/resprot.4072

**Published:** 2015-06-02

**Authors:** Paula M Frew, Saad B Omer, Kimberly Parker, Marcus Bolton, Jay Schamel, Eve Shapiro, Lauren Owens, Diane Saint-Victor, Sahithi Boggavarapu, Nikia Braxton, Matthew Archibald, Ameeta S Kalokhe, Takeia Horton, Christin M Root, Vincent L Fenimore, Aaron M Anderson

**Affiliations:** ^1^ Emory University Department of Medicine, Division of Infectious Diseases Emory University School of Medicine Decatur, GA United States; ^2^ Emory University Department of Behavioral Sciences & Health Education Rollins School of Public Health Atlanta, GA United States; ^3^ Emory University Hubert Department of Global Health Emory University Atlanta, GA United States; ^4^ Texas Woman's University Department of Health Studies Denton, TX United States; ^5^ Emory University Department of Epidemiology Rollins School of Public Health Atlanta, GA United States; ^6^ Colby College Department of Sociology Waterville, ME United States; ^7^ Georgia State University Department of Middle and Secondary Instructional Technology School of Education Atlanta, GA United States; ^8^ Emory University Department of Neurology Emory University School of Medicine Atlanta, GA United States

**Keywords:** health disparities, clinical trials, churches, study recruitment, African Americans

## Abstract

**Background:**

Underrepresentation of older-age racial and ethnic minorities in clinical research is a significant barrier to health in the United States, as it impedes medical research advancement of effective preventive and therapeutic strategies.

**Objective:**

The objective of the study was to develop and test the feasibility of a community-developed faith-based intervention and evaluate its potential to increase the number of older African Americans in clinical research.

**Methods:**

Using a cluster-randomized design, we worked with six matched churches to enroll at least 210 persons. We provided those in the intervention group churches with three educational sessions on the role of clinical trials in addressing health disparity topics, and those in the comparison group completed surveys at the same timepoints. All persons enrolled in the study received ongoing information via newsletters and direct outreach on an array of clinical studies seeking participants. We evaluated the short-, mid-, and longer-term effects of the interventional program on clinical trial-related outcomes (ie, screening and enrollment).

**Results:**

From 2012 to 2013, we enrolled a balanced cohort of 221 persons in the program. At a 3-month follow-up, mean intention to seek information about clinical trials was higher than baseline in both treatment (mu=7.5/10; sigma=3.1) and control arms (mu=6.6/10; sigma=3.3), with the difference more pronounced in the treatment arm. The program demonstrated strong retention at 3-month (95.4%, 211/221) and 6-month timepoints (94.1%, 208/221).

**Conclusions:**

The “Dose of Hope” program addressed an unmet need to reach an often overlooked audience of older African Americans who are members of churches and stimulate their interest in clinical trial participation. The program demonstrated its appeal in the delivery of effective messages and information about health disparities, and the role of clinical research in addressing these challenges.

## Introduction

### Older-Age Racial and Ethnic Minorities Underrepresented in Clinical Research

Underrepresentation of older-age racial and ethnic minorities in clinical research is a significant problem in the United States, as it impedes medical research advancement of effective preventive and therapeutic strategies [1]. In the United States, older minorities (age ≥ 60 years) suffer from greater morbidity and mortality stemming from chronic illnesses (eg, cancer, congestive heart failure, obesity, diabetes, and dementia), as well as from infectious diseases (eg, pneumonia, influenza, and septicemia) [1-6]. Recent studies have corroborated that health disparities arise from attributable differences in socioeconomic status, resulting in an increased burden of illness and disability among African Americans over the age of 65 years [7]. These inequalities in access to care and treatment, a lack of health-supporting social networks, and lack of knowledge about prevention interventions and clinical treatments are also known challenges to the achievement of health equity[8].

Demographic trends point to a significant increase in the both the burden of disease among African Americans in the United States, as well as the proportion of the population that will be African American by 2030 [5]. Older African Americans remain severely underrepresented in clinical trials, despite this population suffering greater prevalence of many diseases than the white population, including greater prevalence of some cancers, cardiovascular disease, diabetes, influenza and pneumonia, obesity, and other conditions such as dementia [9-14]. For example, among the 39,574 patients observed from 17 studies included in a meta-analysis of clinical trial participation for prostate cancer from 1993 to 2011, only 5.3% of participants in the US studies were African American [15]. Older African Americans have similar participation rates (9.9%) in Alzheimer’s disease trials [9]. In two other randomized controlled trials for influenza vaccines, older African American participation was as low as 2.5% (N=48) to 4.9% (N=450) [16,17].

The US Centers for Disease Control and Prevention (CDC) advocates the importance of expanding access to clinical trials and providing information to community members to facilitate study participation by older minorities for whom new prevention and treatment options may be beneficial [4,18]. The 1994 National Institutes of Health mandate specifying the inclusion of women and minorities in federally sponsored studies also underscored the importance of recruiting and retaining diverse racial and ethnic groups to ensure that social justice and scientific aims are achieved through the conduct of research [19]. Enrollment of older persons, including racial and ethnic minorities, in clinical trials is of national interest in effectively addressing health disparities and Healthy People 2020 objectives [1,5,6,20].

The call to increase racial and ethnic minority participation in clinical research has invigorated efforts to identify effective community engagement and recruitment approaches, yet very few interventions have been subjected to rigorous scientific assessment [21-24]. We therefore tested the feasibility of a community-developed faith-based intervention to increase ethnic diversity in clinical research. A “Dose of Hope” engages one of the most powerful forces for community and personal behavioral change in the South, African American communities of faith. The church is a trusted institution in African American communities and has partnered with other organizations to address a variety of health disparity concerns [25-27]. The churches selected for this project had histories of successful partnerships with state and local health departments, local college and universities, and service-oriented community-based organizations, among others.

### Study Implementation and Training

To operationalize this faith-based intervention, we assigned three churches to host the “Dose of Hope” intervention, which included an initial half-day program for congregation members at baseline, followed by two subsequent two-hour small group sessions at three and six months for follow-up. Consensus on program topics and presenters was achieved by holding multiple meetings with pastors, other faith leaders, content area experts, and community advisory board members. Through this approach, content areas selected for delivery included topics such as “Health Disparities in our Community”, “Why Bother with Clinical Trials?”, “The Role of the African American Church in Clinical Research and Human Protections”, “Clinical Trial Updates”, as well as specific health condition presentations (eg, influenza, human immunodeficiency virus/acquired immune deficiency syndrome [HIV]/[AIDS], stroke, and hypertension). For each of these topics, we included background on the issue, its relevancy to the population, direct and indirect health effects, and how community members can get involved to address the concerns. Each session also included time for questions, answers, and up to 10 minutes of group discussion.

As faith leaders were involved in developing and delivering some components of the intervention, they were invited to a small group content development/planning meeting and a separate program delivery training session prior to implementation. We created preliminary slide decks for each session and conducted a crosswalk discussion on standardized talking points. Presenters then rehearsed their talks with study leaders (Principal Investigator, program director, other content area faculty). Prior to delivery at the designated session(s), we followed up with the presenters to ensure that they had adequate training with presentation equipment and educational spaces to ensure that speakers did not experience unnecessary challenges with these logistics.

### Process Evaluation

We sought to identify the factors contributing to intervention success and our participants’ satisfaction in the program. To achieve this, we conducted a comprehensive program evaluation to assess qualitative (eg, attitudes, self-efficacy and empowerment, and network opinions) and quantitative (demographic, housing and insurance status, health care utilization, provider trust, etc) elements contributing to clinical trial enrollment and moderation of the intervention effect. The program evaluation enabled our team to discover the factors that contributed to the program’s success. Understanding what specific program components were effective including messages conveyed, along with the recruitment and retention processes, enabled us to develop “best practices” for community engagement in clinical research.

We implemented an evaluation strategy informed by the CDC framework for public health program evaluation, along with principles drawn from Utilization-Focused Evaluation [28,29]. The CDC framework enabled us to develop our program theory and assess how interdependent components and underlying processes contribute to impact [28] (Table 1).

**Table 1:** CDC evaluation framework for study protocol [28,29].

Step 1: Engaging stakeholders	Through our ongoing work with faith leaders and community partners, we solicited advice on our study instruments to ensure that they were culturally appropriate, and that they elicited important contextual, communication, and network factors that may contribute to clinical trial outcomes. During the project, we also scheduled ongoing discussions with faith leaders at the churches to maintain strong engagement.
Step 2: Describing the program	We developed a “program description” during the first study quarter that included the need for the project, expected effects, activities, resources, stage of development, context, and logic model. This model described the hypothesized mechanism for change underscored by our theoretical orientation, and its potential overall impact on the realization of increased participation in clinical research.
Step 3: Focusing the evaluation design	This aspect focused on assessing “Dose of Hope’s” feasibility for wider dissemination, describing its implementation successes and challenges, and final assessment of the program’s effects.
Step 4: Gathering credible evidence	We collected data on program attributes throughout the intervention period to strengthen the credibility of program findings. Table 1 highlights some of the process indicators relating to participation rates and intervention effects that were collected during the intervention period to bolster the project’s credibility and utility.
Step 5: Justifying conclusions	The final evaluation products, including manuscripts, presentations, reports, and newsletters, reflected the values and efforts of all stakeholders involved in the process. The evidence was continuously synthesized and interpreted with partner agency input, and recommendations are being made on the program’s future via consensus. This process builds on our previous experience with the “Dose of Hope” program evaluation in which we considered its format, delivery, sustainability, and potential for scale-up expansion.
Step 6: Ensuring use and sharing lessons learned	The evaluations for the “Dose of Hope” pilot endeavors were very useful for identifying problems and implementation challenges, along with opportunities and advances. In addition to our planned internal use, we intended to broadly disseminate the findings from this program to others who could benefit from the lessons learned from our community-participatory research model.

## Methods

### Community Based Participatory Research Approach

The “Dose of Hope” program utilized the Community Based Participatory Research approach to develop the intervention. Our project incorporated “best practice strategies” including building trust with the community, hiring community members as lead staff, and delivery of cultural competency training to reinforce the knowledge and skills necessary to work with this special group [8].

### Protocol Aims

The study addressed two major aims. First, we examined the effect of the educational intervention on clinical trial recruitment of older African Americans by tracking screening and enrollment outcomes for 24 months following the pilot study. Within this scope, we explored the pathways through which individual and network factors operated to shape enrollment differences. We also sought to characterize participants’ health-supporting network linkages.

To evaluate the feasibility and potential effectiveness of “Dose of Hope”, we used a cluster randomized controlled trial design to test whether delivery of a three-session group intervention increased the proportion of older African Americans who enroll in an array of chronic and infectious disease-related clinical trials. The sampling frame of about 20 churches was identified via ethnographic observation and key informant interviews. Given our intervention’s goal of increasing clinical trial participation rates among older African Americans, eligibility for the project venues was restricted to: (1) faith organizations with congregation membership of ≥ 30% African Americans ages ≥ 50 years, and (2) faith organizations situated within one of the 22 counties comprising metropolitan Atlanta. Prospective venues were matched by denomination and estimated congregational membership. From the 20 in the sampling frame, six churches were randomly selected to participate in the study using matched-pair randomization.

**Figure 1:**
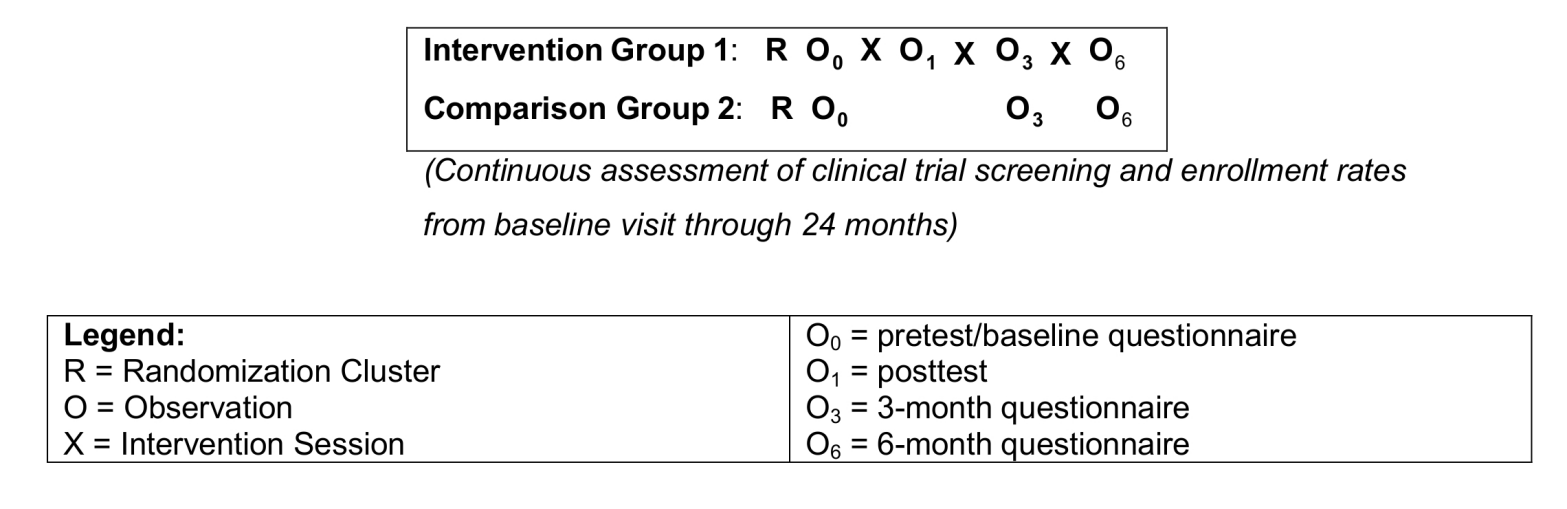
Delivering a "Dose of Hope” study design.

Using random selection, one church in each pair was allocated to the intervention condition (Intervention Group 1) and the matched pair was assigned to the control condition (Comparison Group 2). Figure 1 shows the study design.

Participants completed intermediate assessments at 3 months and 6 months, and were followed longitudinally for 24 additional months. Through monthly telephone and email outreach, all participants from both intervention and control arms were notified of local study opportunities and screened for specific clinical trials by recruiters. “Dose of Hope” partnered with 23 other studies/clinical programs in 2013 and 2014 to provide recruitment and referral opportunities to program participants. The studies that participants were referred to addressed a variety of health disparities to encourage “Dose of Hope” participants to join studies of great interest to them. These included clinical studies on how the brain controls head movements; sleep, memory, and ageing; urinary symptoms; vaccine trials including those for influenza (H7N9), yellow fever, cholera, and pneumonia; Alzheimer's disease; experiences of intimate partnership violence; treatment for trauma from domestic/interpersonal violence; post traumatic stress disorder; HIV risk factors; treatment of fatigue related to breast cancer treatment; the effect of high blood pressure medication on thinking ability and brain activity; insomnia and blood pressure; language processing; and heart failure and memory.

### Church Sampling Strategy

We drew a list of all prospective churches from those that agreed to participate as either intervention or control sites. All sites on the roster were enumerated. We then drew from this enumerated sampling frame of nearly 20 churches, choosing the 6 churches that ultimately participated as sites for this study. For each site selected, we also identified one back-up church in the event that the selected church was no longer able to participate. The three denominations represented were the American Methodist Episcopalian (AME), Baptist, and Seventh-Day Adventist (SDA) practices. Following venue randomization, we worked with faith leaders to identify specific days of the week and time blocks that were ideal for producing an adequate pool of potential participants that could be recruited from the selected churches. We worked directly with the churches to outreach to target populations within their church, drawn from elders’ groups and bible study groups, and via direct outreach via flyers, presentations at church gatherings, and other activities. We then worked directly with the church contacts assigned to our study to conduct research activities at each location; it was during those specified times that we conducted our research activities with their approval.

### Participant Recruitment

Within the selected churches, we screened and enrolled persons recruited through outreach conducted by pastors, health ministers, and other congregational leaders. Church members included those who regularly attend services, tithe, may be active on church committees, and attend church-related social gatherings. Eligibility requirements for the study included: (1) self-identified race/ethnicity as black or African American; (2) age 50 or older; (3) residing in the 22-county metropolitan Atlanta region; (4) plans to reside in Atlanta for 12 months following recruitment; (5) no previous history of participation in clinical research studies; (6) church-confirmed congregant status; and (7) ability to read and write English.

Our initial aim was to enroll a minimum of 105 participants on each arm, of whom 35 were to be selected from each intervention site. However, given the potential for significant attrition among an older cohort, we determined that oversampling the cohort would retain sufficient power to evaluate the primary endpoint of clinical trial enrollment in the event of a ≥20% loss of participants during the course of the study. Thus, the recruitment efforts resulted in 11 more persons than the original target of 210 persons (final N=221 participants).

Study team members screened all participants for eligibility. Screened members of intervention churches who were not eligible based on personal criteria were given the opportunity to attend the sessions and given the same program materials as enrolled participants, but they did not complete questionnaires at the sessions. Those who were ineligible from control churches were provided with information about clinical research study opportunities; they did not complete any questionnaires. All persons deemed eligible to participate underwent the informed consent process prior to the start of the first session.

### Intervention Groups

The program for intervention participants included initial breakout sessions facilitated by faith leaders and clinical researchers that covered the following issues specified in the facilitator’s guidebooks: “Myths about Clinical Trials”, “Challenges to Research Involvement”, “Benefits and Rewards to Involvement in Clinical Trials”, and “Hope in the Community”. Subsequent to the half-day seminar hosted at each intervention church, two follow-up small group sessions (one hour each) were scheduled with 12-20 persons to occur at 3- and 6-month timepoints. Disease-related topics included HIV/AIDS, influenza vaccination, stroke, diabetes, hypertension, diet and nutrition, and others. Participants also discussed structural determinants of health such as the role of violence, incarceration, food availability, health care access, housing, employment, discrimination, and transportation. Session leaders also engaged study participants in deep discussions about the lack of community participation in prevention and treatment studies, navigating social support influences, and safeguards to ensure participant safety and well-being. All elements of the sessions were determined prior to implementation to ensure standardized delivery of the program at all locations. A program manual and standard operating procedures was developed to ensure fidelity.

Control participants did not receive any type of study intervention, but completed the study questionnaires at baseline, 3-month, and 6-month timepoints. We invited control participants to attend other events such as community health fairs, screenings, and educational presentations on health topics unrelated to clinical trials (eg, mammography screening). Both control and intervention participants received quarterly newsletters informing them about the study activities, preliminary results and study findings, and clinical trial opportunities.

### Measures

The three key endpoints are captured by screening, enrollment, and referral pattern tracking of all participants with data provided by study coordinators who are following our cohort. Additionally, we gathered self-reported data from surveys at all three timepoints as longitudinal measures of intention to screen and enroll in clinical studies, and at 3- and 6-months asked participants to report any studies that they had screened and enrolled in so that we could conduct verifications of these events with study coordinators. We also tracked the number of persons who screened (captured as an outcome), but were determined to be “medically ineligible” for specific studies due to comorbidities and health conditions. These events enable us to understand “screen failures” within the cohort due to specific conditions and other study enrollment placement. Thus, people are consistently referred to a number of studies to capture screening and enrollment rates. With multiple referrals made to studies, this often resulted in multiple screening and enrollment events.

This study built upon our previous theoretical research drawing upon micro and macro-theoretical approaches to understand community engagement in clinical research [30-32]. We targeted three major outcomes from this program: clinical trial screening, enrollment, and promotion/diffusion of participation messages. We also focused on reinforcing positive social norms and attitudes toward clinical trial participation, as is consistent with the major components of the Theory of Reasoned Action. We created an expanded Theory of Reasoned Action (TRA), which incorporated Diffusion of Innovations (DOI) constructs [33,34]. The key domains of TRA (ie, attitudes and social norms) were addressed in all aspects of the program [30,35]. For example, facilitators engaged participants in an interactive discussion on attitudes and beliefs toward clinical trials (ie, “Why Bother with Clinical Trials?”), dealing with negative social appraisal of involvement (ie, “Inspiration, Information, and Motivation to Act”), and forming behavioral intentions (ie, “Clinical Trial Update: Progress in Drug Development and Prevention Research”). The “breakout sessions” enabled a deeper level of conversation, as we targeted attitudes and behavioral beliefs, along with social norms, with this intervention. We built upon TRA to promote more favorable attitudes toward clinical trial participation by reinforcing positive norms toward community engagement in health research.

In addition, our measures drew upon DOI to explore the dimensions of social networks to understand how certain ideas and behaviors become socially acceptable, and therefore become more commonplace in communities [33]. We incorporated social network analyses to elicit underlying social network processes driving clinical research participation (ie, homophilous or heterophilious communication among caregivers, social support systems, and others). These measures enabled our team to explore the impact of interconnectedness on trial-related outcomes. This effort ultimately advanced our understanding of the intersection of networks and community-level factors that influence participatory outcomes [36-39].

Table 2 details questionnaire domain measures for all participants. Our measures were reliable and valid [32,40]. Given the large volume of survey data collected at each timepoint, we used self-administered pen-and-paper surveys developed at a sixth-eighth grade reading level. We believe this also reduced interviewer bias. In addition, up to 10 participants were asked to do intercept interviews which are typically ≤ 10 minutes to gauge what they learned, what they intend to do as a result of their participation, and their satisfaction with the program.

**Table 2:** “Dose of Hope” measures.

Variables			
**Baseline**			
	Sociodemographics:	Gender, age, educational attainment, marital and sexual orientation status, employment and housing status, income level, health care utilization and insurance status	Behavioral/community characteristics/indicators (TRA and DOI) [30-35,40] Faith and place of worship affiliation, community affiliations, group memberships, mono and polymorphic network opinion leadership, network linkages, volunteerism experience, previous clinical trial experiences and knowledge, recent volunteer health behaviors (eg, organ donation in past 12 months), health research and clinical trial attitudes, social support indicators (eg, RAND social health battery) and personal network interactions, trust in provider, health research organization, and clinical research involvement scales (measured by CRIS)
**Follow-up (3- and 6-month)**			
	Sociodemographics:	Employment and housing status, health care utilization, and insurance status (3-month recall)	Behavioral/community characteristics/indicators (TRA and DOI) [30-39] Health and clinical trial information and media consumption, modes of network communication, community affiliations and group memberships, network linkages, opinion leadership, clinical trial interest and knowledge, Emory clinical research experience, recent volunteer health behaviors (eg, organ donation in past 3 months), health research and clinical trial attitudes, perceived social support for clinical research involvement (personal network support), social activism congruence, trust in provider scale (measured by CRIS)
Clinical trial variables (24-months post baseline)			Clinical trial variables (tracking screening and enrollment database) Date of clinical trial site contact, date of screening, date of enrollment, reasons for study exclusions, recruitment source/venue
	

### Three- and Six-Month Follow-Up Questionnaires

The three-month and six-month follow-up questionnaires included measures repeated from the baseline survey to explore longitudinal changes. We also planned to randomly select up to 20 persons to participate in 30-45 minute interviews on their experiences in the program and intentions and behaviors post intervention. These interviews enabled us to gather information on their knowledge, attitudes, beliefs, intentions, and screening and enrollment behaviors. We concluded interviews as soon as the data were determined to be saturated, meaning that no new content was arising as a result of these discussions. This resulted in a total of 31 persons interviewed during and after the program.

All data, including personal identification numbers, from participants that enroll in Emory clinical studies were linked back to their previous questionnaires. The information contained in the clinical trial database includes sociodemographics, contact data, recruitment source, motivations for participation, and enrollment outcomes including reasons (if known) for exclusion (eg, health reason and age).

### Data Analysis

Based on our past experience with trial enrollment, we assumed a baseline trial enrollment rate of 6% in the control group. We sought a minimal sample size of 210 individuals (105 in each arm) by sampling 3 clusters with at least 105 subjects in the intervention group and 3 clusters with 105 subjects in the control group. This would enable us to achieve 80% power to detect a difference between the group proportions of at least 15% (ie, 21% of individuals in the intervention group) using the two-sided Z test (unpooled) at a 5% significance level. Sample size estimates were adjusted for expected intracluster correlation and an expected retention rate of 80%.

We examined differences in demographic and behavioral variables across the intervention and control groups with a combination of *t* tests, chi-square, and linear mixed models. In addition to bivariate models, multivariate analyses were performed adjusting for demographic, behavioral, and contextual variables. These analyses included calculation of the increase between timepoints in mean scores for intention to seek information about clinical trials and intention to join clinical trials. We selected the linear mixed model approach as most appropriate for the longitudinal analyses, as they account for clustered design. In addition, the study protocol enabled us to assess the impact of the study intervention on clinical trial enrollment events using Poisson regression. For the primary analysis, incidence rate ratios included counts of individuals enrolled in clinical trials during the 24-month follow-up period from the intervention arm compared to the counts of enrolled individuals who were recruited from the control arm. All models account for clustering at the church level, and therefore robust confidence intervals are most appropriate.

## Results

### Early Descriptive and Longitudinal Study Results

Early descriptive and longitudinal results from this ongoing study indicate successful recruitment of the “Dose of Hope” cohort (Table 3). The two study arms were well balanced (n=109 in control and n=112 in intervention), as was denominational participation. Participants were primarily female (78.2%, 173/221), though men were also represented. Ages ranged from 50 to over 90, with nearly half of the participants’ ages 60 to 69 (48.8%, 108/221). The distribution of age differed significantly between the intervention and control group (Mann-Whitney *U* test; *P*=.03). Almost half were currently married or living with a domestic partner (46.1%, 102/221), yet many were divorced or separated (26.6%, 59/221) or widowed (15.8%, 35/221). Only a few persons had never been married (10.9%, 24/221).

### Participant Sociodemographics

We recruited an educated population. The majority of participants had attained a technical or associate's degree (29.9%, 66/221) or high school/GED or less (35.7%, 79/221) as their highest level of educational attainment. Most participants were retired or unemployed (67.0%, 148/221), and one-quarter had annual household income less than twenty thousand dollars per year (27.6%, 61/221). Half of the participants were insured through managed care or a combination of private insurance and managed care (50.2%, 111/221), one-third used private insurance only (33.0%, 73/221), and 10.0% were uninsured (22/221). The intervention and control groups did not vary significantly with respect to any of the other variables presented in Table 3 (chi-square test; *P*>.05).

### Participant Retention

We observed a very strong retention rate over time. The program had better-than-expected 3-month (95.4%, 211/221) and 6-month retention rates (95.0%, 208/220, accounting for the loss of one person who died during the project from a nonrelated study cause). We believe that the qualitative and evaluative data will provide insight on what components of the program participants felt were most valuable and deserving of their dedicated time in the sessions. The social network analyses may also provide additional perspective on the extent of social cohesion experienced by participants in this program as a motivator to continue participation, and will help determine whether the social-educational nature of the program may have fostered its strong retention.

### Early Results

Early results assessing intentions to seek clinical trial information, screen, and enroll reflect a moderate amount of change over baseline. Participants' self-reported intentions to seek information about and to join clinical trials are summarized in Tables 4 and 5. At baseline, participants expressed relatively neutral opinions about their likelihood to contact researchers about clinical trials, balanced in both the intervention (mu=5.7/10; sigma=2.9) and control (mu=5.5/10; sigma=2.9) arms. Yet at 3 months, mean intention to seek information about clinical trials was higher than baseline in both treatment (mu=7.5/10; sigma=3.1) and control arms (mu=6.6/10; sigma=3.3), with the difference more pronounced in the treatment arm. Mean intention to seek information decreased slightly at 6 months in both arms. A similar trend is present in participants' intention to join a clinical trial, though less pronounced than with intention to seek information about clinical trials.

**Table 3:** Sociodemographic characteristics of study participants (N=221).

Sociodemographic characteristics		n	%
**Study arm**			
	Control	109	49.3
	Intervention	112	50.7
**Church denomination**			
	AME	60	27.1
	Baptist	78	35.3
	SDA	83	37.6
**Gender**			
	Male	48	21.7
	Female	173	78.3
**Age (years)**			
	50-59	62	28.1
	60-69	108	48.9
	70-79	41	18.6
	80-89	5	2.3
	90+	2	0.9
	Missing	3	1.4
**Marital status**			
	Single/never married	24	10.9
	Married/domestic partner	102	46.2
	Divorced/separated	59	26.7
	Widowed	35	15.8
	Other	1	0.5
**Educational attainment**			
	High school/GED or less	79	35.7
	Technical associate’s degree	66	29.9
	Bachelor’s degree	37	16.7
	Master’s/doctorate	39	17.6
**Employment**			
	Unemployed or retired	148	67.0
	Employed (part-time and full-time)	65	29.4
	Missing	8	3.6
**Annual household income (US dollars)**			
	Less than 20,000	61	27.6
	20,001-40,000	49	22.2
	40,001-60,000	36	16.3
	60,001-80,000	20	9.0
	80,001-100,000	19	8.6
	More than 100,000	13	5.9
	Missing	23	10.4
**Medical insurance policy**			
	No insurance/other	22	10.0
	Private insurance plan	73	33.0
	Managed care/combination plan	111	50.2
	Missing or don't know	15	6.8

**Table 4:** Intention to contact Emory about clinical trials in next 6 months (N=221).

	Mean intention to seek information^a^		
	Baseline	3-month^b^	6-month^b^
Intervention	5.7	7.5	7.1
Control	5.5	6.6	6.5

^a^ measured on 10-point Likert scale

^b^ Participants who had screened at 3 or 6 months were given an intention score of 10 (5 at baseline, 14 at 3-month, 15 at 6-month).

**Table 5:** Intention to join a clinical trial in next 6 months (N=221).

	Mean intention to join trial^a^		
	Baseline	3-month^b^	6-month^b^
Intervention	5.8	6.2	6.3
Control	5.8	5.9	5.7

^a^ measured on 10-point Likert scale

^b^ Participants who had screened at 3 or 6 months were given an intention score of 10 (5 at baseline, 14 at 3-month, 15 at 6-month).

### Participant Intent to Enroll in Clinical Trials

Self-reported initiation of contact and joining of clinical trials at the 3- and 6-month timepoints is summarized in Tables 6 and 7. After 3 months, a relatively large proportion of participants reported contacting researchers about clinical trial participation, including a large proportion of both the intervention (37.5%, 42/112) and control (32.1%, 35/109) arms. A slightly smaller proportion indicated current contact with researchers at the 6-month timepoint (30.6%, 34/111 in the intervention arm and 26.6%, 29/109 in the control arm). A smaller proportion of participants passed through the study screening stage to be deemed eligible to participate in clinical trials. After 3 months, (6.2%) 7/112 of the intervention and (1.8%) 2/109 of the control participants had joined clinical trials, moving up to (9.0%) 10/111 of intervention and (2.8%) 3/109 of control participants after 6 months.

**Table 6:** Participants contacting researchers about clinical trial participation (3-month total = 221; 6-month total = 220).

	Number of participants/arm n (% of arm)	
	3-month, n (%)	6-month, n (%)
Intervention	42/112 (37.5)	34/111 (30.6)
Control	35/109 (32.1)	29/109 (26.6)

**Table 7:** Participants joining clinical trials (3-month total = 221; 6-month total = 220).

	Number of participants/arm n (%)	
	3-month, n (%)	6-month, n (%)
Intervention	7/112 (6.2)	10/111 (9.0)
Control	2/109 (1.8)	3/109 (2.7)

## Discussion

### Community Engagement in Clinical Trials

Community engagement involves multidirectional communication for the overarching purpose of enhancing the public’s trust in the effort. Evidence-based methods include consultation, dialogue, and collaboration with communities [41] to develop shared understanding and meanings associated with the research programs [2]. This process also fosters the community’s voice in research endeavors and develops a sense of community empowerment [3]. These methods are vital for reaching minority communities and women to sustain their involvement in medical research studies [4,5,8,9,12], and to promote favorable health outcomes in the population [10,11].

Therefore, the role of community engagement in clinical research figures prominently in addressing salient concerns among diverse groups. Previous findings related to clinical trial recruitment of African Americans suggest differences exist among men and women from diverse communities in their motivations for participation [42,43]. The early results from this study suggest the value of a strong researcher-participant relationship, particularly for female participants, in which study volunteers are made to feel comfortable, are treated well, and share rapport and good communication with the study team members [42]. Moreover, women appreciate notification of research conducted in their locales, and of its importance and relevance to their communities. The results from this study suggest that the program, which was successful in recruiting a cohort comprised of mostly women, successfully created a positive environment aligned with these factors.

Researcher involvement in the local community also is a significant motivator in clinical trial participation [43]. This is an important factor that merits commentary for this protocol. Not only was the research team comprised of health scientists and physicians deeply committed to the project, but they also had histories of working on other projects with the selected churches. Given the ill-fated history of the Tuskegee syphilis study involving African American men, and its resonance with older African Americans, we were conscientious of the potential for lower levels of trust in their assessment of health care providers and health care systems [44,45]. Moreover, we recognized that negative experiences and perceived bias in this population’s previous health care encounters likely influenced their trust of us as part of the medical establishment [46,47].

To address these concerns, we set out to build a community-based participatory action model that would enable participants to have direct experiences with the “Dose of Hope” providers in the sessions. By leveraging the role of the church in its ability to send persuasive "social cues", we were able to build greater trust in providers and medical entities involved in the program [41,44,48]. Although there are similarities observed across studies, clinical trial perceptions vary greatly among African American communities. Variations may be due to socioeconomic status, access to care, health service utilization patterns, insurance provision, and interpersonal dynamics of patients and physicians [44,47]. With this assemblage of factors, interpersonal and perceived socioenvironmental normative messaging may have had an influential effect on clinical trial decision-making.

With the 1994 National Institutes of Health (NIH) mandate specifying inclusion of women and minorities in research, greater emphasis has been placed on recruiting and retaining these populations. Although minorities are not participating in health research at a level equal to whites [41,43,48,49], it is important to recognize that knowledge of and access to health research activities may have a favorable impact on willingness to participate in health research [18]. In a large scale review study of 70,000 persons, minorities were found to be more willing to participate in clinical and surgical studies than whites [18]. These findings indicate that little difference is seen in enrollment patterns when minorities are invited to participate in health research studies [18]. With these differences taken into account, the authors conclude that underrepresentation in health research is likely due to other factors.

Recent evidence on minority participation in health research indicates a desire for information of the research activity in the community, greater demand to understand the relevance of the research efforts in addressing medical problems, and occasions to learn about clinical research entities and study volunteer participation [19,39]. Thus, the creation of opportunities to serve these needs is a necessary precursor for effective community engagement with African American communities. These aforementioned reasons provide rationale for the creation of our program.

### Future Directions

We will evaluate the indirect effect of our intervention for the enrollment-related endpoint. This will be achieved by comparing the counts of individuals enrolled in clinical trials who belonged to the intervention churches, but did not attend the small group sessions versus counts of individuals enrolled who belonged to the control churches and were not included in the control arm (ie, were not included in the baseline data collection). For this analysis, an offset term comprising of the total number of congregation members in each type of church is typical. The total (ie, direct and indirect) effect of the intervention can be assessed by including all individuals belonging to either arm and by using an offset term of the total number of congregation members in each arm.

We are also conducting social network analysis per protocol. Network data were collected at the 6-month timepoint. We asked people to respond to 4 items that asked them to name the top 3 persons involved in the program (eg, health minister, pastor, study team member, and others involved) that they would turn to for advice in life, about their health, about personal crises, and with whom they socialize. This information provided us with an understanding of the extent of homophily within groups, and it helped us to determine whether network density impacted message diffusion [36,39]. By gathering name-based information, we were able to capture the extent of integration of the program “actors” (study staff, speakers) with church member participants, and the degree of reciprocity evoked by bringing information to participants and their willingness to give back in the relationship [36-38].

### Conclusions

The “Dose of Hope” program is a feasible, sustainable, and engaging model for education and recruitment of older African Americans in faith-based settings. The early results of this study indicate that the program had an effect on intentions to seek clinical trial information, and in the longer term, participate in appropriate studies. Additionally, the strong retention rate of the cohort suggests that the program was well received by participants. “Dose of Hope” may therefore usher in a new model for clinical trial engagement of willing, yet overlooked, diverse participants.

## Abbreviations

AIDS: acquired immune deficiency syndrome
AME: American Methodist Episcopalian
CDC: Centers for Disease Control and Prevention
DOI: Diffusion of Innovations
HIV: human immunodeficiency virus
NIH: National Institutes of Health
SDA: Seventh-Day Adventist
TRA: Theory of Reasoned Action

## Appendix

Multimedia Appendix 1 NIH study section reviewer comments.
