# Supplementary material for: Delivering a “Dose of Hope”: A Faith-Based Program to Increase Older African Americans’ Participation in Clinical Trials
Source: JMIR Res Protoc. 2015 Jun 2;4(2):e64. doi: 10.2196/resprot.4072 (PMC4526899; doi:10.2196/resprot.4072)
Supplement: Multimedia Appendix 1 [file resprot_v4i2e64_app1.pdf]

1R03AG042831-01 Frew, Paula

RESUME AND SUMMARY OF DISCUSSION: This highly significant application will assess and characterize a faith-based intervention to increase representation of older African Americans in clinical research. If successful, this research could likely inform strategies to improve recruitment and retention of minorities in clinical trials. The investigators are a strong team and a good match for the project with a stellar publication record. During the discussion the panel noted several strengths of the application, including: the strong research design, the strong preliminary studies, the teams familiarity with using similar techniques for recruitment in prior research, and the strong community partnerships. Overall this was considered an impressive application from an exceptional investigator and enthusiasm was extraordinarily high. There are no major weaknesses in this extremely strong application and reviewers concurred that the impact of this research on the field would likely be high.

DESCRIPTION (provided by applicant): This project presents a unique opportunity to evaluate a community based culture-centered program to increase older (ages e 55 years) black/African Americans' participation in clinical research. "A Dose of Hope," will engage one of the most powerful forces for community and personal behavioral change in the South, African American communities of faith. Few interventions designed to increase racial and ethnic minority participation in clinical research have been subjected to rigorous scientific evaluation let alone those tailored for older Americans. Therefore, a comprehensive assessment of the multifaceted "Dose of Hope" intervention will inform the development, implementation, and evaluation of community-developed interventions on a national level. This project will enroll 2 matched cohorts, of 105 volunteers each, drawn from 6 representative churches across the Atlanta metropolitan area resulting in a sample of 210 participants. Churches will be matched on selected characteristics including denomination type and membership size. The intervention consists of a half-day program with facilitated small group discussions at 3- and 6-month timepoints. The intervention groups will participate in activities that promote knowledge about minorities and their historical experiences in clinical research, group harms, contemporary clinical trial ethics and regulations, and opportunities for community involvement in clinical research. The control groups will receive general printed material about clinical research. All participants will undergo baseline assessment with follow up at 3- and 6- month intervals. The primary outcome of the study will be the difference in proportions of volunteers in the intervention and control groups who enroll in a clinical trial at one-year post-intervention. The results of our study will fill a critical gap in rigorously tested approaches to increase the numbers of older racial and ethnic minorities in clinical trials which will help address health disparities, and will further our understanding of the influence of social networks on clinical trials participation.

PUBLIC HEALTH RELEVANCE: Participation of older black/African Americans in clinical research studies is much lower compared to whites despite the fact that the group is disproportionately impacted by many chronic, degenerative, and infectious diseases. "A Dose of Hope" was created to increase diversity and participation in research trials through engagement with black/African American communities of faith. Our goal is to understand if the "Dose of Hope" program is effective in achieving greater enrollment of older blacks in clinical studies, and what elements of the program specifically contribute to the realization of greater enrollment of this group.

CRITIQUE 1:

Significance: 1
Investigator(s): 1
Innovation: 1
Approach: 1

Environment: 1

Strengths

- Addresses a major problem with implications for all research with older adults, that is, the ability to recruit adequate samples of African Americans and eventually other minorities
- Uses key informant community participants to help formulate the components of the intervention
- Is based on prior experience using similar techniques for recruitment

Weaknesses

- None noted.

1. Significance:

Strengths

- Just about anything that would boost the participation of minorities in clinical trials would be a significant contribution to health science

Weaknesses

- None noted.

2. Investigator(s):

Strengths

- Research team has much experience conducting and analyzing similar community-based efforts and publishing the results

Weaknesses

- None Noted.

3. Innovation:

Strengths

- Works with a new intervention that could have a big payoff

Weaknesses

- None Noted.

4. Approach:

Strengths

- Uses key informant community participants to help formulate the components of the intervention
- Is based on prior experience using similar techniques for recruitment

Weaknesses

- None noted.

5. Environment:

Strengths

- A very supportive environment for carrying out the research

Weaknesses

- None Noted.

Protections for Human Subjects:

Acceptable Risks and/or Adequate Protections

- acceptable

Data and Safety Monitoring Plan (Applicable for Clinical Trials Only):

Acceptable

- o acceptable

Inclusion of Women, Minorities and Children:

G1A - Both Genders, Acceptable

M2A - Only Minority, Acceptable

C3A - No Children Included, Acceptable

- intentionally studies minorities

Vertebrate Animals:

Not Applicable (No Vertebrate Animals)

Biohazards:

Not Applicable (No Biohazards)

Budget and Period of Support:

Recommend as Requested

CRITIQUE 2:

Significance: 2
Investigator(s): 2
Innovation: 2
Approach: 2
Environment: 2

Overall Impact: This project addresses not only a minority population but also a program focused on faith to increase older adults' participation in research. Program can be replicated at other minority health focused locations in addition to faith based locations.

1. Significance:

Strengths

- Proposed meeting people where they gather and participate in programs is good.
- Outreach to a minority population not ordinarily participating in research trials is very appropriate and will contribute to the literature.

Weaknesses

- None Noted.

2. Investigator(s):

Strengths

- Researchers are qualified and competent.

Weaknesses

- None Noted.

3. Innovation:

Strengths

- Intertwining important gathering site for participants with health related emphasis provides greater opportunity to attract and retain participants. Built on successful program with younger persons involved.

Weaknesses

- None Noted.

4. Approach:

Strengths

- Realistic and well thought out concerning the population to be served. Built on successful program with younger community-based persons which bore relevant outcomes.

Weaknesses

- None Noted.

5. Environment:

Strengths

- Site that is comfortable and reachable for participants. There is trust established.

Weaknesses

- None Noted.

Protections for Human Subjects:

Acceptable Risks and/or Adequate Protections

Data and Safety Monitoring Plan (Applicable for Clinical Trials Only):

Not Applicable (No Clinical Trials)

Inclusion of Women, Minorities and Children:

G1A - Both Genders, Acceptable

M2A - Only Minority, Acceptable

C3A - No Children Included, Acceptable

Vertebrate Animals:

Not Applicable (No Vertebrate Animals)

Biohazards:

Not Applicable (No Biohazards)

Budget and Period of Support:

Recommended as requested.

CRITIQUE 3:

Significance: 2
Investigator(s): 1
Innovation: 1
Approach: 2
Environment: 1

Overall Impact: The problem of increasing minority participation in clinical trials is a highly important public health issue. The investigator has a good track record of doing similar research projects. The community intervention approach is an innovative, evidence based approach to addressing this problem. The research design and the data analysis plans are well developed. The university environment and the support of the local churches is strong.

1. Significance:

Strengths

- The problem addressed, participation of minorities in clinical trials, is a highly significant issue for researchers and also a pressing public health concern in view of pervasive health disparities in minority populations.

Weaknesses

- None Noted.

2. Investigator(s):

Strengths

- The investigator is well qualified to lead the project with a track record of similar research with other health problems in other populations.

Weaknesses

- None Noted.

3. Innovation:

Strengths

- The community intervention being tested is an innovative approach to changing attitudes of older minority participants toward participation in research.
- The use of churches as the base for delivery of the intervention is innovative.

Weaknesses

- None Noted.

4. Approach:

Strengths

- The design of the study is quite strong including matching of types of churches and randomization of the intervention.
- The analysis plan is well developed
- The mix of quantitative and qualitative methods is a strength for this research topic

Weaknesses

- None Noted.

5. Environment:

Strengths

- The research environment is strong and supportive
- There is strong support from area churches for the project

Weaknesses

- None Noted.

Protections for Human Subjects:

Acceptable Risks and/or Adequate Protections

- Risks are low for this social science research project. Good protections are in place for confidentiality.

Data and Safety Monitoring Plan (Applicable for Clinical Trials Only):

Not Applicable (No Clinical Trials)

Inclusion of Women, Minorities and Children:

G1A - Both Genders, Acceptable

M2A - Only Minority, Acceptable

C3A - No Children Included, Acceptable

Vertebrate Animals:

Not Applicable (No Vertebrate Animals)

Biohazards:

Not Applicable (No Biohazards)

Budget and Period of Support:

Recommended as requested.

THE FOLLOWING RESUME SECTIONS WERE PREPARED BY THE SCIENTIFIC REVIEW OFFICER TO SUMMARIZE THE OUTCOME OF DISCUSSIONS OF THE REVIEW COMMITTEE ON THE FOLLOWING ISSUES:

PROTECTION OF HUMAN SUBJECTS (Resume): ACCEPTABLE

INCLUSION OF WOMEN PLAN (Resume): ACCEPTABLE

INCLUSION OF MINORITIES PLAN (Resume): ACCEPTABLE

INCLUSION OF CHILDREN PLAN (Resume): ACCEPTABLE

COMMITTEE BUDGET RECOMMENDATIONS: The budget was recommended as requested.

+ Derived from the range of percentile values calculated for the study section that reviewed this application.

NIH has modified its policy regarding the receipt of resubmissions (amended applications). See Guide Notice NOT-OD-10-080 at http://grants.nih.gov/grants/guide/notice-files/NOT-OD-10-080.html.
The impact/priority score is calculated after discussion of an application by averaging the overall scores (1-9) given by all voting reviewers on the committee and multiplying by 10. The criterion scores are submitted prior to the meeting by the individual reviewers assigned to an application, and are not discussed specifically at the review meeting or calculated into the overall impact score. For details on the review process, see http://grants.nih.gov/grants/peer_review_process.htm#scoring.
